# Supplementary material for: The structural basis of lipid scrambling and inactivation in the endoplasmic reticulum scramblase TMEM16K
Source: Nat Commun. 2019 Sep 2;10:3956. doi: 10.1038/s41467-019-11753-1 (PMC6718402; doi:10.1038/s41467-019-11753-1)
Supplement: Supplementary file 1 — Supplementary Information [file 41467_2019_11753_MOESM1_ESM.pdf]

Supplementary Information

The structural basis of lipid scrambling and inactivation in the endoplasmic reticulum scramblase TMEM16K

S. R. Bushell, A.C.W. Pike, M. E. Falzone et al.,

Supplementary Figures

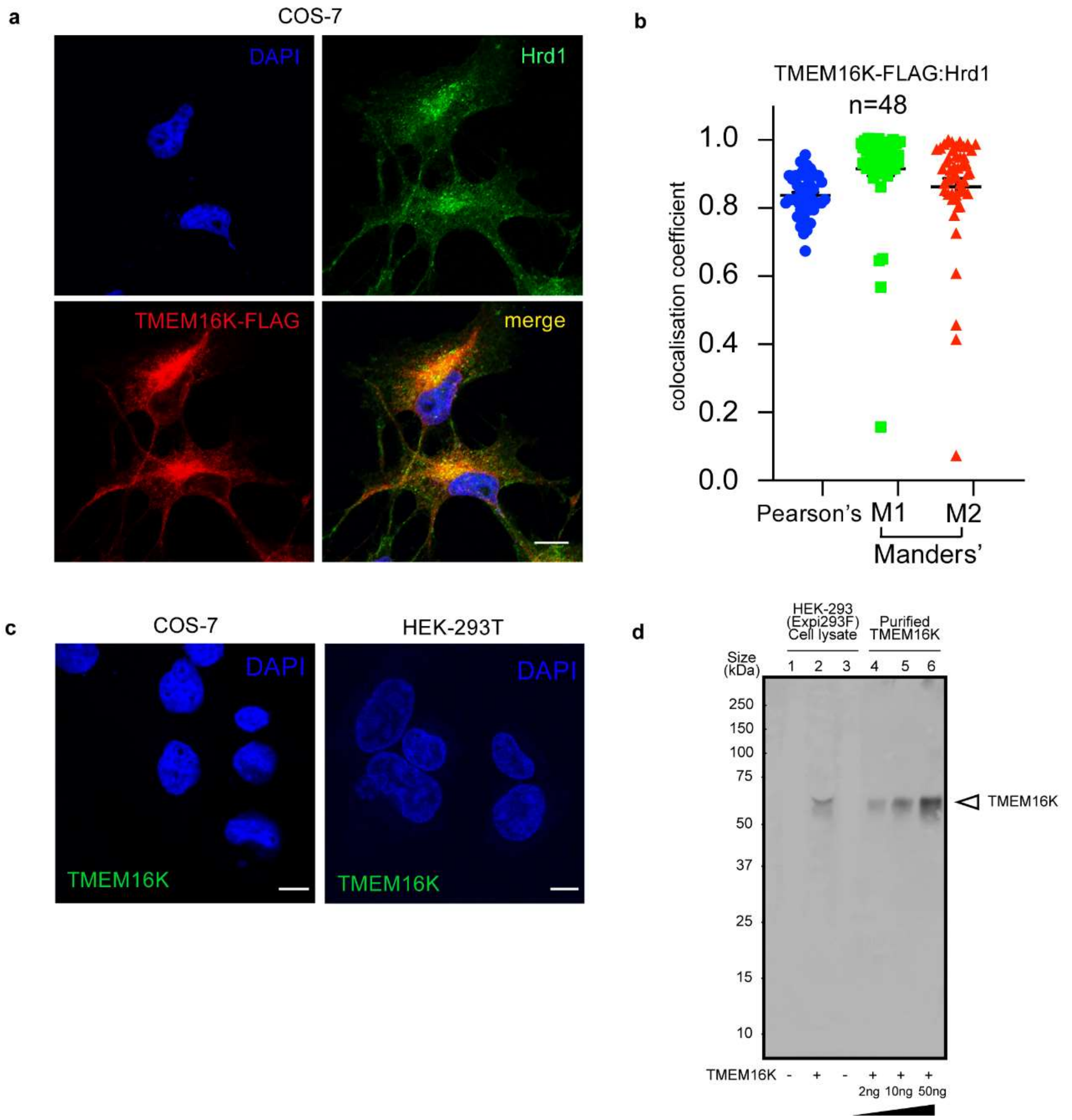

Supplementary Figure 1. Cellular localisation of TMEM16K. a Representative confocal images of COS-7 cells expressing TMEM16K-TEV-His$_{10}$-FLAG and immunostained with anti-FLAG (TMEM16K, red), Hrd1 (green), along with DAPI staining of nuclei (blue) and the merge. Scale bars = 10 µm; magnification: 63x. **b** Quantitative analysis of TMEM16K-TEV-His$_{10}$-FLAG and Hrd1 colocalization in COS-7 cells as determined by Pearson's and Manders' overlap coefficients (M1, M2). Mean and s.e.m. are shown, n=48 **c** Representative merged confocal images of untransfected HEK-293T and COS-7 cells stained with anti-TMEM16K (green) and DAPI (blue) to confirm TMEM16K antibody specificity. Scale bars = 10 µm; magnification: 63x. Quantifications of cellular localisation (Pearson's and Manders' coefficients) are listed in Supplementary Table 1. **d** Western Blot demonstrating specificity of the Anti-TMEM16K antibody. Lane 1-2: Lysate from BV-transduced HEK-293 (Expi293F) cells expressing an (1) unrelated membrane protein or (2) hTMEM16K. Lane 3: Cell lysate from non-transduced expi293F cells. Lane 4-6: Purified TMEM16K, expressed in expi293F cells at 2 ng, 10 ng and 50 ng. Note: Like many membrane proteins, TMEM16K migrates faster in an SDS-PAGE gel, (equivalent to a molecular weight of approximately 55 kDa) than suggested by its actual size (79 kDa for the monomer). Source data are provided as a Source Data file.

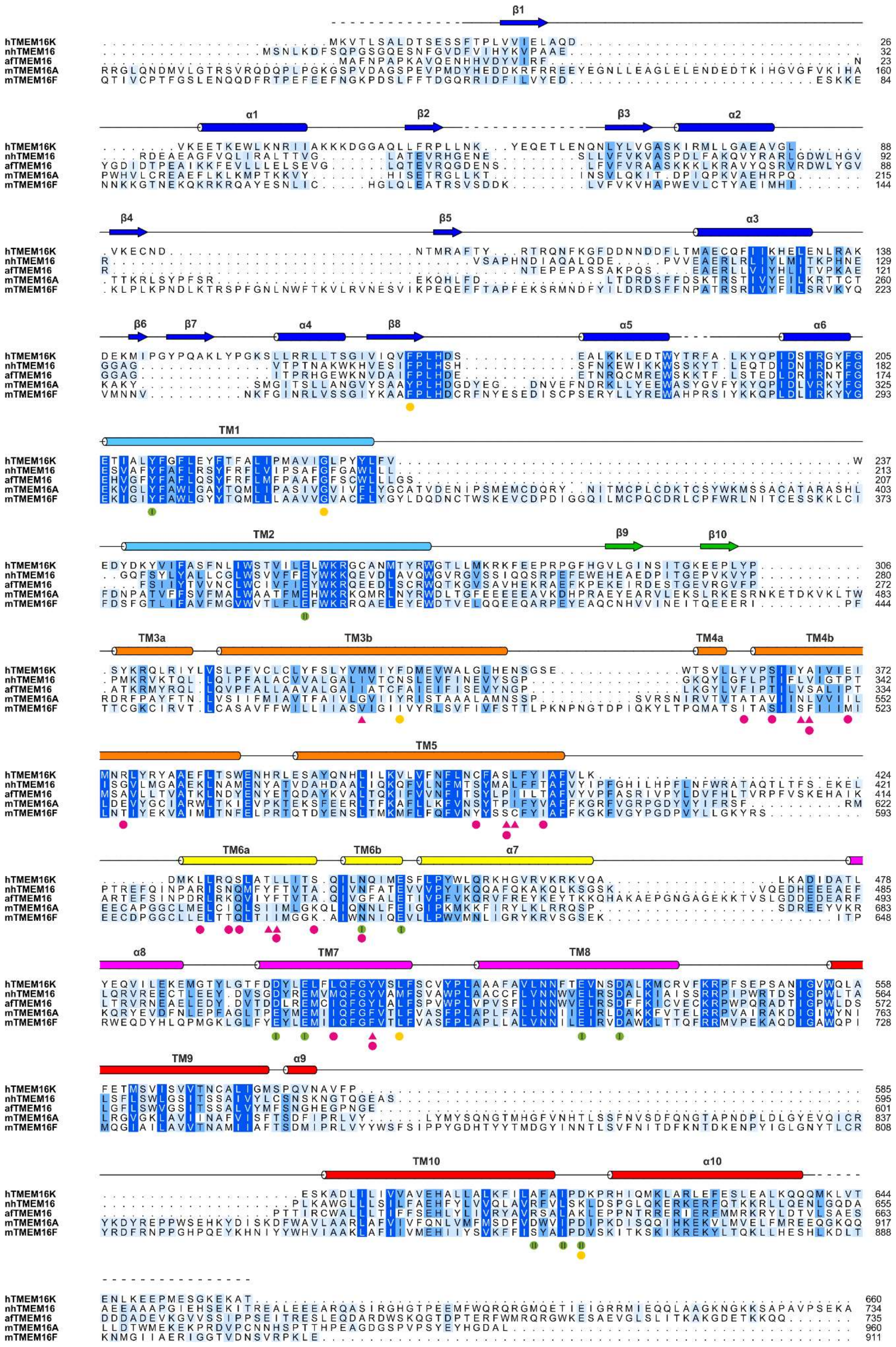

β1
hTMEM16K
nhTMEM16
afTMEM16
mTMEM16A
mTMEM16F
α1
β2
β3
α2
β4
β5
α3
β6
β7
α4
β8
α5
α6
TM1
TM2
β9
β10
TM3a
TM3b
TM4a
TM4b
TM5
TM6a
TM6b
α7
α8
TM7
TM8
TM9
α9
TM10
α10

Supplementary Figure 2. Structure-based sequence alignment of human TMEM16K (UniProt: Q9NW15, ANO10_HUMAN), mouse TMEM16A (Q8BHY3, ANO1_MOUSE), mouse TMEM16F (Q6P9J9, ANO6_MOUSE) and TMEM16 homologs from fungal species *Nectria haematococca* and *Aspergillus fumigatus*. Conserved and partially conserved residues are highlighted in dark blue and light blue respectively. Residues involved in TM6-8 Ca^{2+}-binding site and TM10/α10 Ca^{2+}-binding site are marked with green dots. Known disease mutations are marked with orange dots. Residues located in the scramblase groove that were identified as forming contacts with lipids in MD simulations are shown as magenta circles and residues in the neck region are shown as magenta triangles. Secondary structure annotation is derived from the LCP TMEM16K crystal structure and are coloured as described in Fig. 3b.

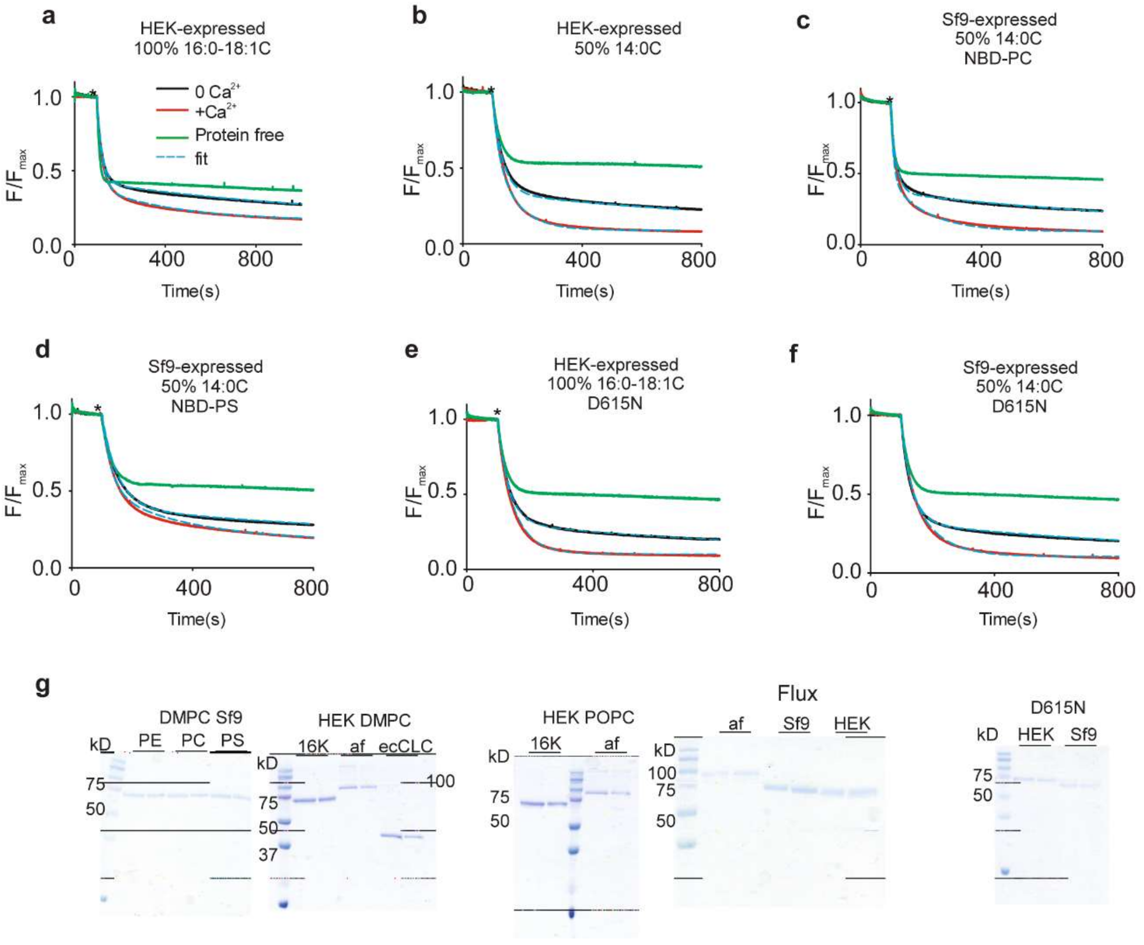

Supplementary Figure 3. Time courses and expression studies for the scramblase assays. **a-f**, Representative time courses of the dithionite-induced fluorescence decay in liposomes made from 100% 16:0-18:1C acyl chains with HEK-expressed TMEM16K **a** 50% 14:0C acyl chains with Sf9-expressed TMEM16K with NBD-PC or NBD-PS **b-c** 50% 14:0C acyl chains with HEK-expressed TMEM16K-D615N **e** or 50% 14:0C acyl chains with Sf9-expressed TMEM16K-D615N **f** with protein-free liposomes (green), TMEM16K proteoliposomes with 0.5 mM (red) or without (black) Ca^{2+}. NBD-PE was used unless otherwise noted. **g** SDS-PAGE of TMEM16K proteoliposomes that were used for scrambling or flux assays. Samples were taken after the first biobead incubation (left) and after the final one (right). All conditions show comparable amounts of TMEM16K at the end of the reconstitution. Source data are provided as a Source Data file.

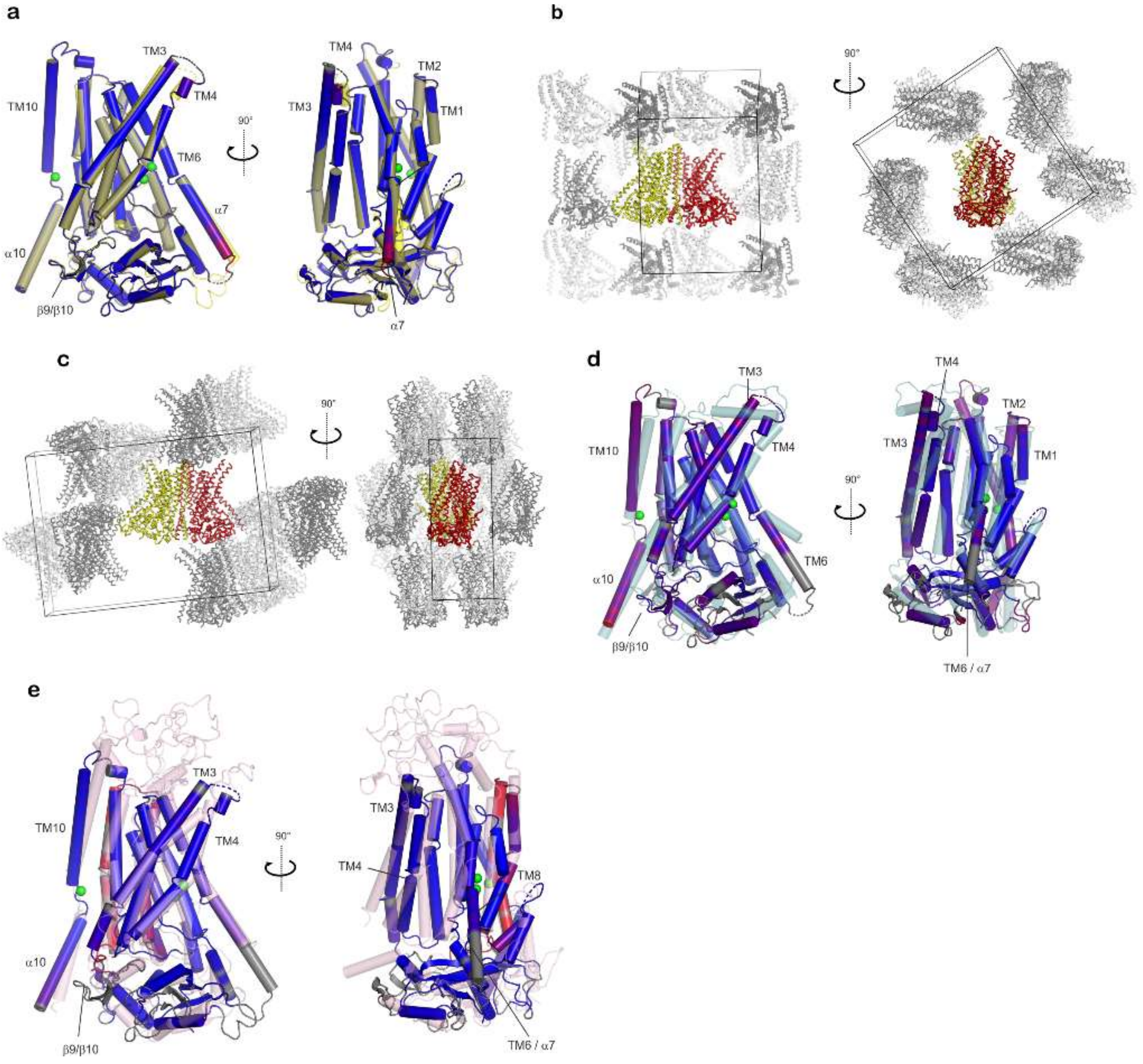

Supplementary Figure 4. Superpositions of TMEM16K with various homologs and crystal packing. a Superposition of TMEM16K crystal structures. The monomer from the vapour diffusion derived crystal structure is coloured as semi-transparent gold. The LCP monomer is coloured by the root mean square differences between the structures, with blue representing those most closely aligned and red the most distant. Bound calcium atoms are depicted as green spheres. **b-c.** Crystal packing within **b** vapour diffusion and **c** LCP crystal forms. TMEM16K is depicted as yellow (chain A) and red (chain B) Cα traces. The unit cell is depicted as a black cube, with symmetry mates depicted as light and dark grey Cα traces. For clarity, some symmetry mates which obscure view of the central molecule are not shown, though all crystal contacts are represented in the figure. **d-e** Superposition of TMEM16K (coloured by RMS deviation as described in Supplementary Figure **4a**, with **d** nhTMEM16K, coloured white, or **e** mTMEM16A, coloured gold. Bound calcium in the TMEM16K structures depicted as green spheres.

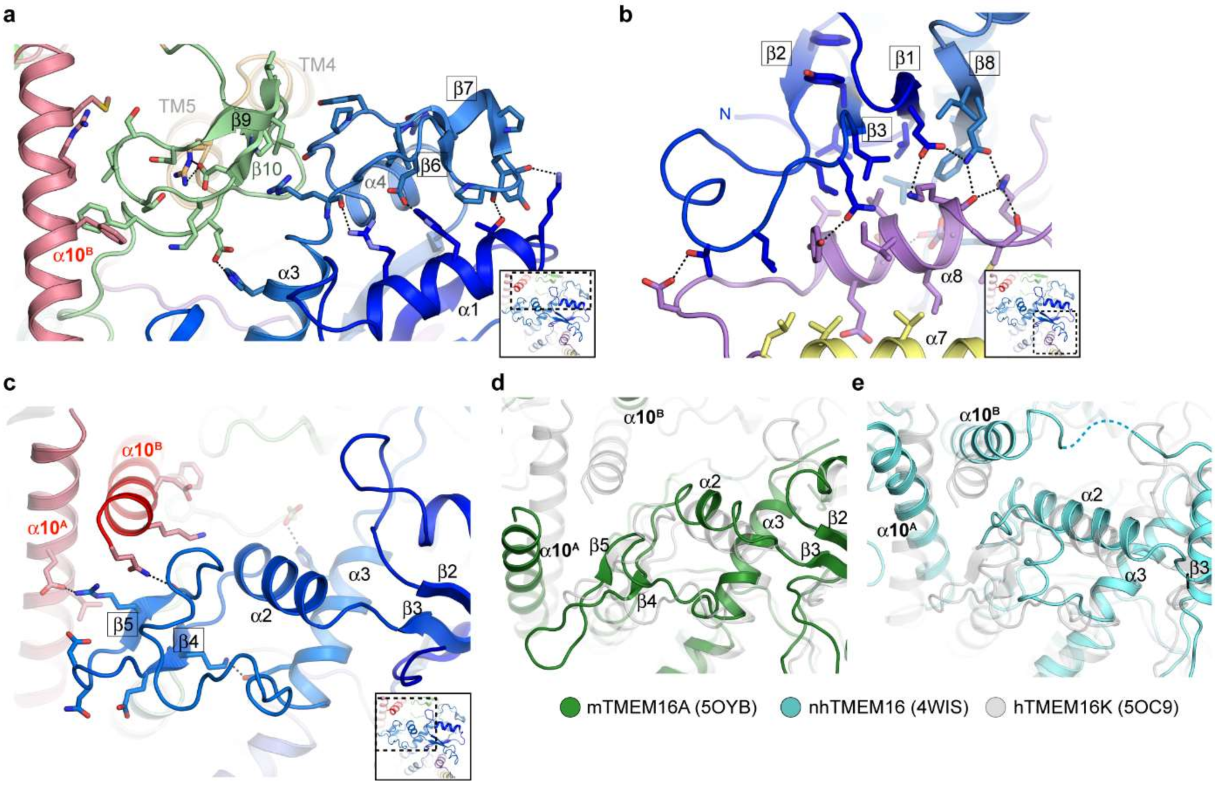

Supplementary Figure 5. Key interactions between the TMEM16K N-terminal cytoplasmic domain (NCD) the TM domain extensions and α10. a β6-β7 hairpin and the associated loops interactions with the β9-β10 hairpin on the TM domain. **b** four stranded β-sheet (β1-β3, β8) is tightly backed against α8, part of an α-helical hairpin formed by α7 and α8, which are extensions of TM6 and TM7. **c** Between β3 and α3 there is a region where the TMEM16K, mTMEM16A and nhTMEM16 structures differs significantly, TMEM16K having a relatively short α3 and a β4-β5 hairpin that interacts directly with the C-terminal $\alpha10^A$ and $\alpha10^B$ helices. **d-e** Corresponding region in (**d**) mTMEM16A (green) and (**e**) nhTMEM16 (cyan).

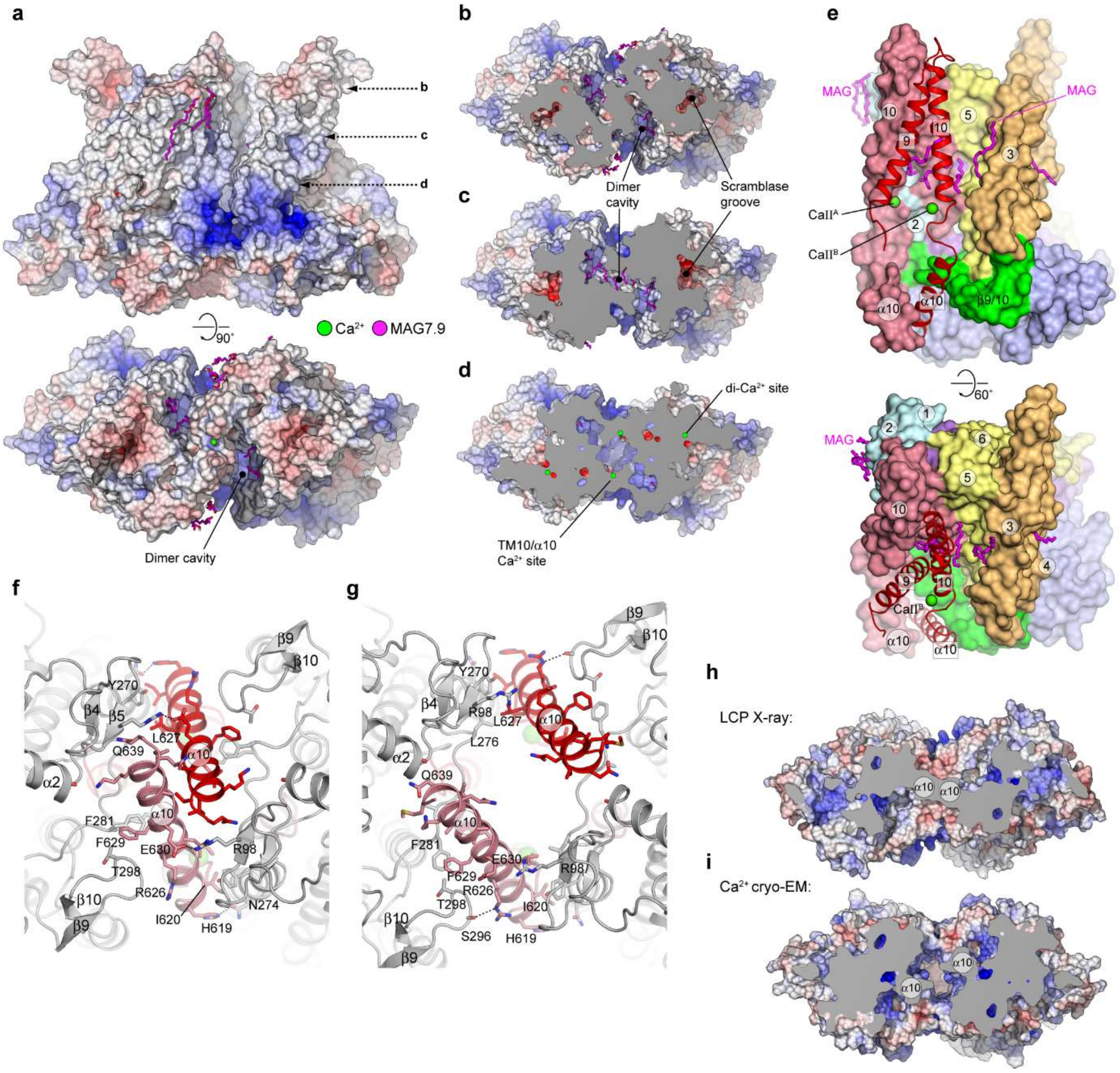

Supplementary Figure 6. The TMEM16K dimer interface and the TM10-α10 Ca $^{2+}$-binding site. a Molecular surface for TMEM16K LCP X-ray structure coloured by electrostatic potential (+/- 10kT/e). **b-d** Sliced molecular surface viewed from ER luminal face showing dimer interface cavity. Approximate locations of sliced view indicated in **a. e** Cut-through of TMEM16K's dimer interface. Chain A is represented as a space-filled model with TM9-10 and α10 from Chain B depicted as ribbon. The TM10/α10 calcium binding sites $CaII^A$ and $CaII^B$ are derived from Chains A and B respectively and calcium ions are depicted as green spheres. MAG lipids are coloured magenta. **f-g** Comparison of the interactions of α10 region of dimer interface for the (**f**) LCP X-ray and (**g**) 2 mM Ca^{2+} cryo-EM structures. The dimer interface is viewed from the cytoplasm. **h-i** Sliced molecular highlighting considerable reduction in the closed scramblase's dimer interface due to repositioning of α10 helices. Molecules are viewed from the cytoplasmic face.

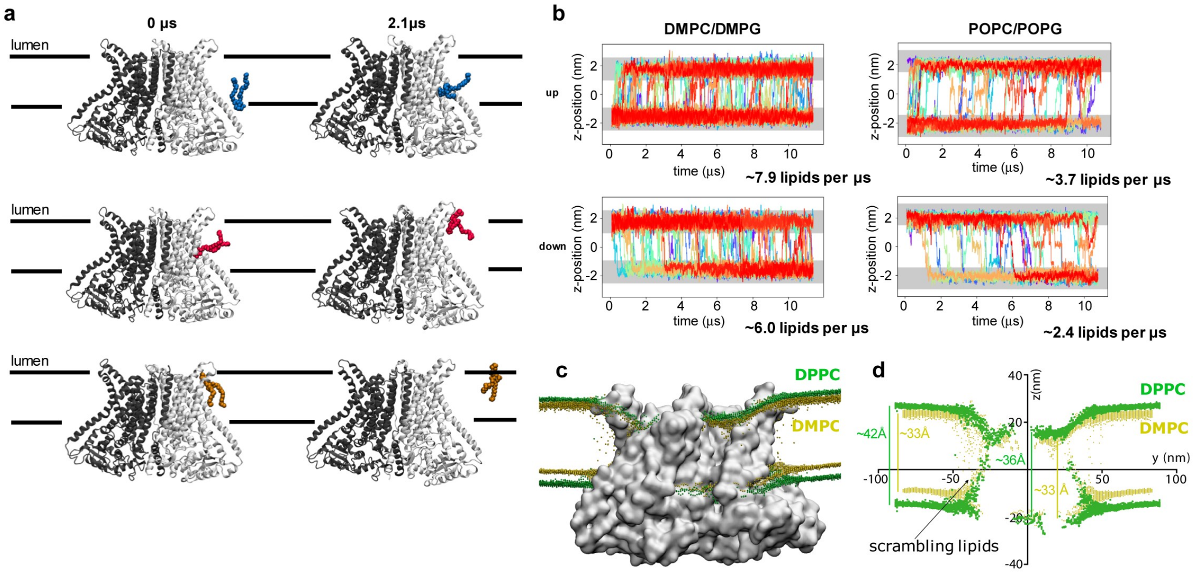

Supplementary Figure 7. MD simulations show lipid scrambling, effects of lipid chain length and membrane thinning. a Snapshots of TMEM16K from atomistic MD simulations, with three lipids shown as coloured beads to highlight the scrambling process. On the left is the position of the lipids at the start of the simulation, and on the right is their positon after 2.1 µs of simulation. Together, these snapshots demonstrate that the full lipid scrambling pathway is sampled. Note that several additional scrambling lipids have been removed from the images for clarity. **b** Lipid scrambling rates as sampled by CG simulation. Plots as per Figure 5 b. The shorter tail DM lipids appear to scramble much faster than the PO lipids, with reported rates calculated as scrambling events / (simulation time). **c** View of TMEM16K from CG simulation data, with the protein shown as white surface, and the average position of the phosphate head group beads shown as coloured points: green for simulations in a DPPC membrane, and gold for in a DMPC membrane. Note that the points have been slabbed in front of and behind TMEM16K for clarity. **d** Positions of the points in panel c plotted as functions of y and z axis. The average distance between phosphate groups in the bulk membrane is ca. 3.3 nm for DMPC and 4.2 nm for DPPC, but around the protein the DPPC thins to ca. 3.6 nm.

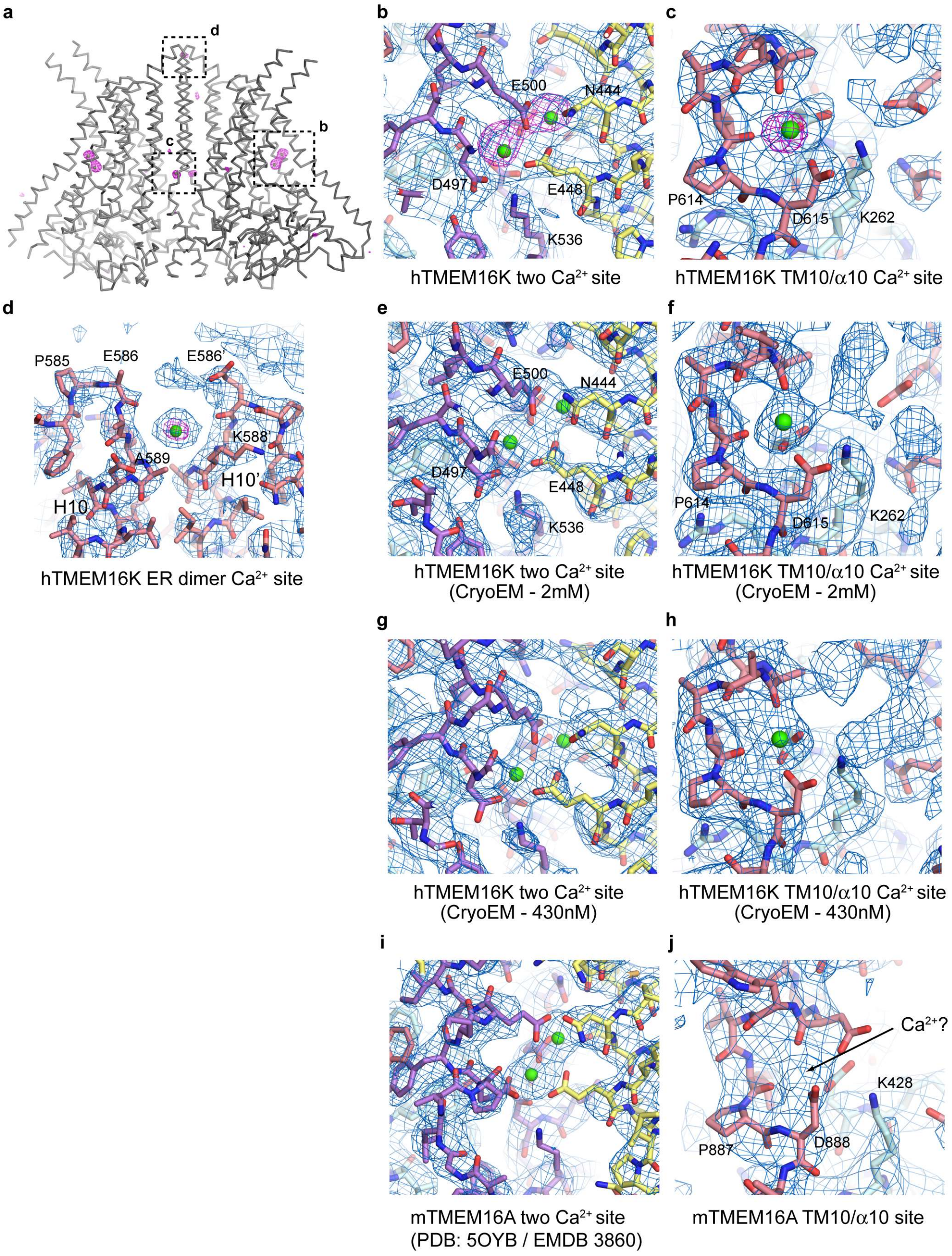

Supplementary Figure 8. Electron density / electric potential maps at the Ca^{2+} binding sites in TMEM16K and TMEM16A. a PHASER single anomalous diffraction log-likelihood gradient (LLG) map (magenta mesh, contoured at 4.5 σ) calculated from data collected at a wavelength of 1.65Å and overlaid on a Cα trace of the final LCP crystal structure. Clear peaks are observed for all assigned Ca^{2+} atoms. **b-c,** Detailed view of **b** two Ca^{2+} site **c** TM10-α10 Ca^{2+} binding site from the LCP crystal structure. Final BUSTER 2FoFc electron density (blue mesh) contoured at 1σ and PHASER LLG map (magenta mesh, contoured at 5.5σ) overlaid on final refined model. **d** Fourth Ca^{2+} ion located on the dimer axis between TM10s on the ER surface of the membrane, found only in the LCP crystal structure. LLG density map (magenta mesh) is contoured at 4σ. **e-f** Corresponding density in the 3.5 Å 2 mM Ca^{2+} cryo-EM map around **e** the TM6-8 two Ca^{2+} and **f** TM10 / α10 Ca^{2+} site. The map is contoured at 3σ. **g,h** Corresponding density in the 4.2 Å 430 nM Ca^{2+} cryo-EM map around **e** the TM6-8 two Ca^{2+} and **f** TM10 / α10 Ca^{2+} site. The map is contoured at 3σ. **i-j** Cryo-EM maps around **i** the two Ca^{2+} site and **j** the TM10-α10 region for the 3.75Å mouse TMEM16A structure (PDB: 5OYB / EMDB 3860). The map is contoured at 7.5σ.

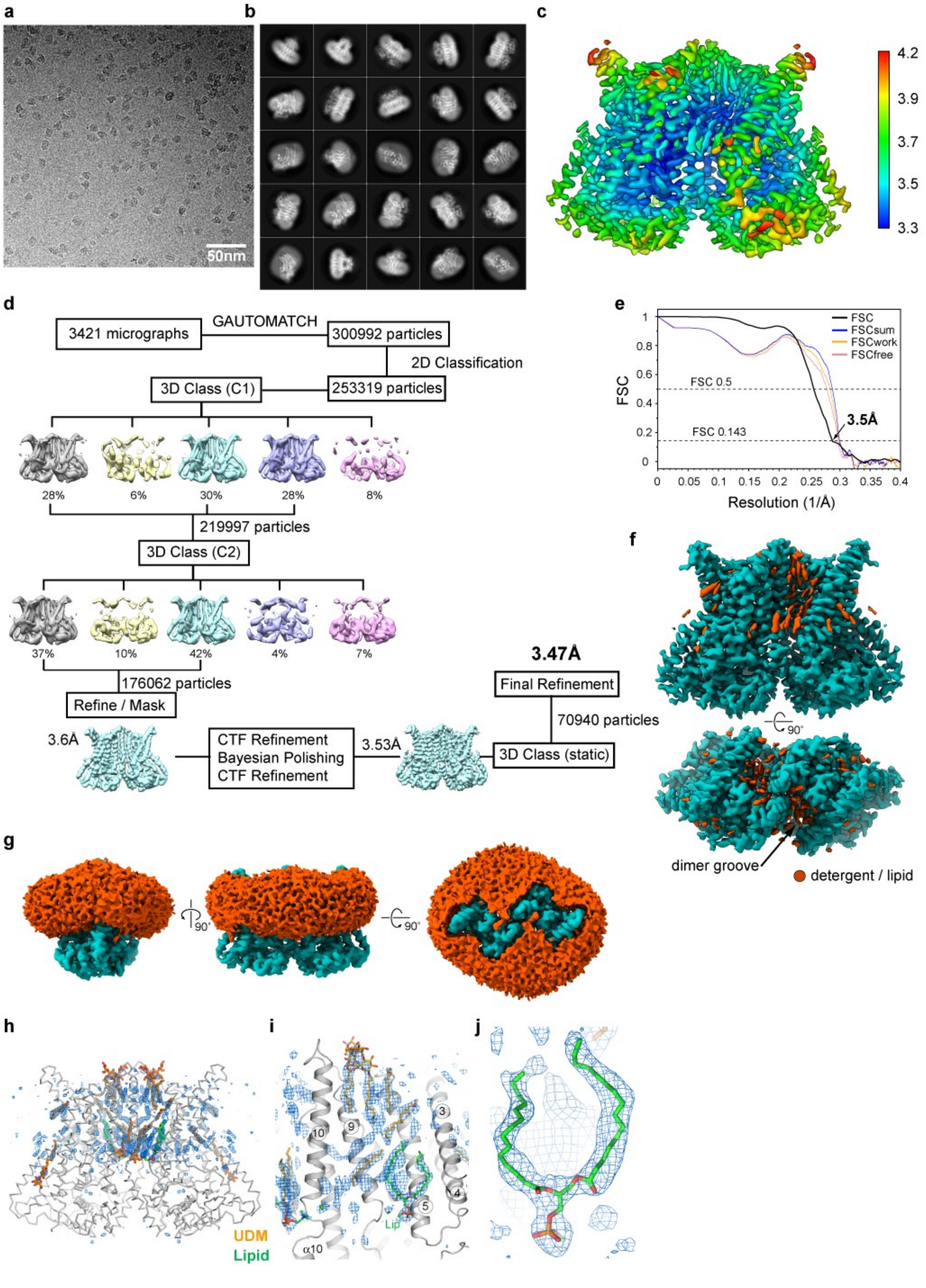

Supplementary Figure 9. Cryo-EM structure determination of TMEM16K in the presence of 2 mM Ca^{2+}. a Representative micrograph. **b** Representative 2D class averages (228px box). **c** Local resolution map calculated using RELION. **d** Workflow for data processing in RELION. **e** Gold standard FSC plot for RELION masked half maps (FSC) and map to model FSC (FSCsum, FSCwork, FSCfree) for refined model as calculated by

phenix.mtriage. **f** Protein (cyan) and detergent/lipid (orange) cryo-EM density. The dimer groove is filled with a large amount of ordered detergent/lipid. **g** .Detergent distribution around dimer. **h** Density and models for UDM detergent molecules and a lipid in the 3.5Å cryo-EM structure. **i** density for a lipid and UDM in the dimer cavity and **j** density for an individual lipid in the cavity on the cytoplasmic leaflet side of the membrane.

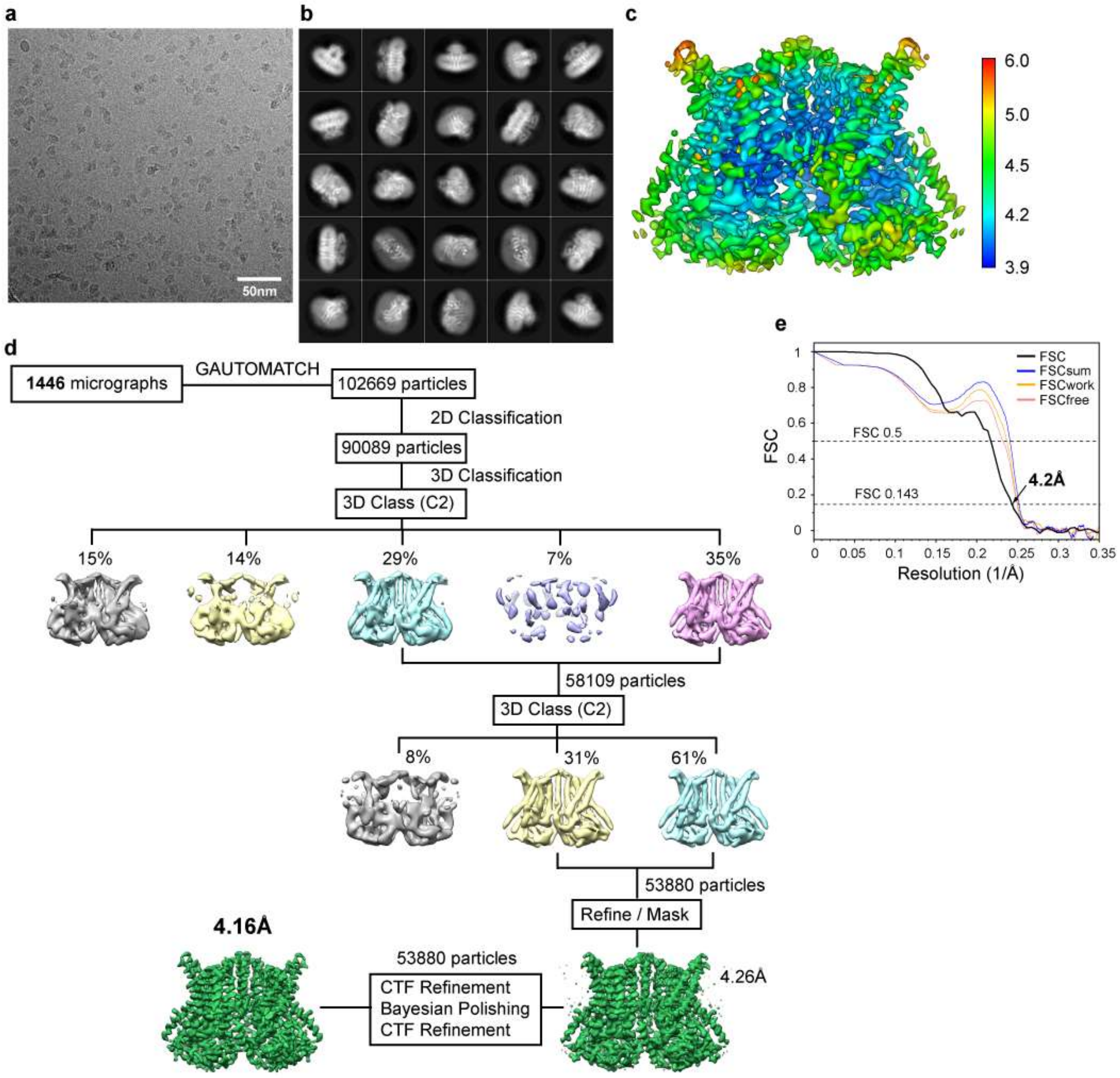

Supplementary Figure 10. Cryo-EM structure determination of TMEM16K in the presence of 430 nM Ca^{2+}. a Representative micrograph. **b** Representative 2D class averages (228px box). **c** Local resolution map calculated using RELION. **d** Workflow for data processing in RELION. **e** Gold standard FSC plot for RELION masked half maps (FSC) and map to model FSC for refined model as calculated by phenix.mtriage.

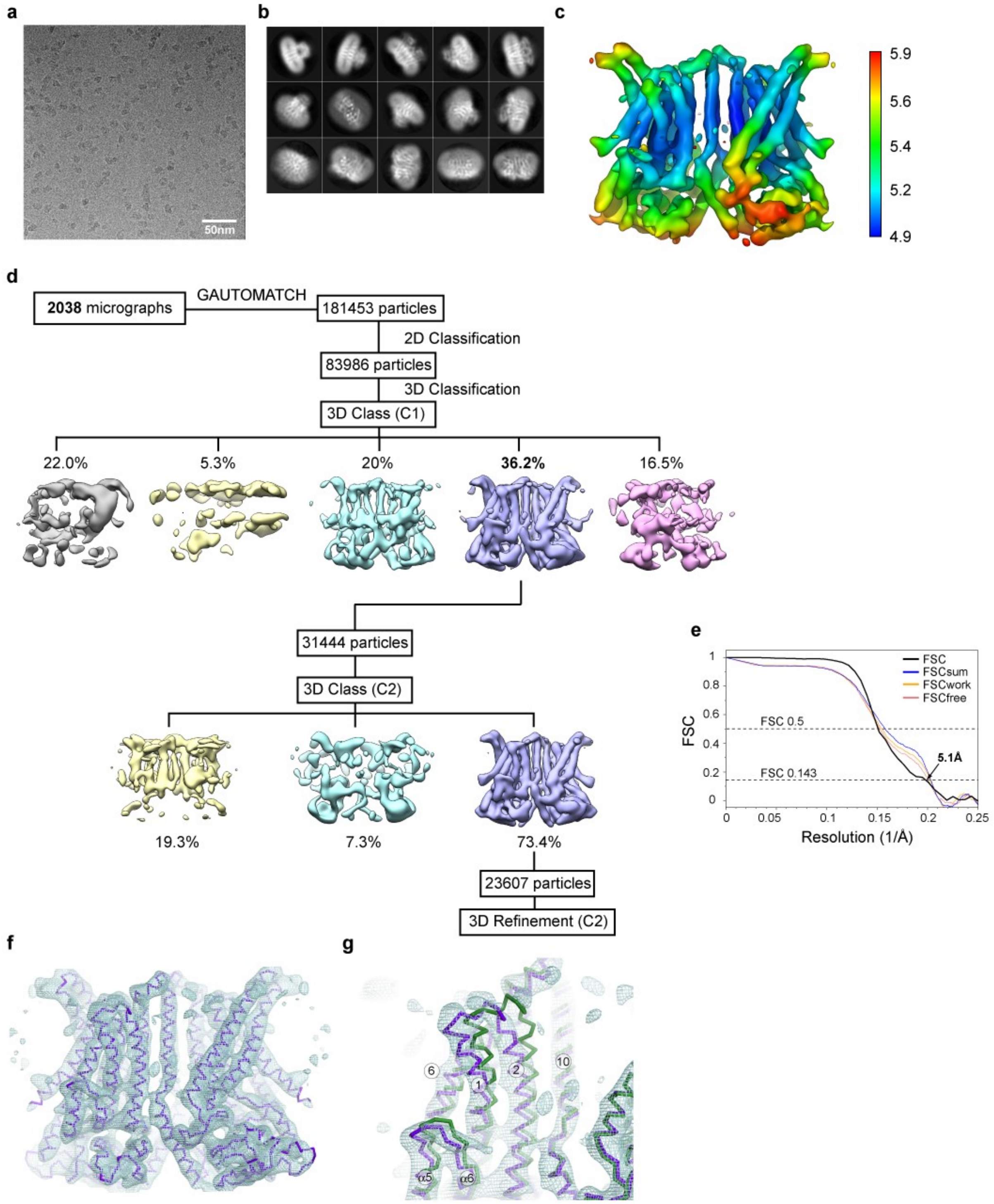

Supplementary Figure 11. Cryo-EM structure determination for Ca^{2+}-free / EGTA TMEM16K. **a** Representative micrograph. **b** Representative 2D class averages (200px box). **c** Local resolution map calculated using RELION3. **d** Workflow for data processing in RELION. **e** Gold standard FSC plot for RELION masked half maps (FSC) and model to model FSC for refined model as calculated by phenix.mtriage. **f** 5.1Å Cryo-EM map (contoured at 3.2σ) overlaid on Cα trace for refined model. **g** Zoomed view of map showing conformational changes in TM1/2, α5-α6 loop region, TM6 and TM10. The Cα trace for the Ca^{2+}-free (purple) and 2 mM Ca^{2+} (green) structures are overlaid on the Ca^{2+}-free cryo-EM map (contoured at 3.7σ).

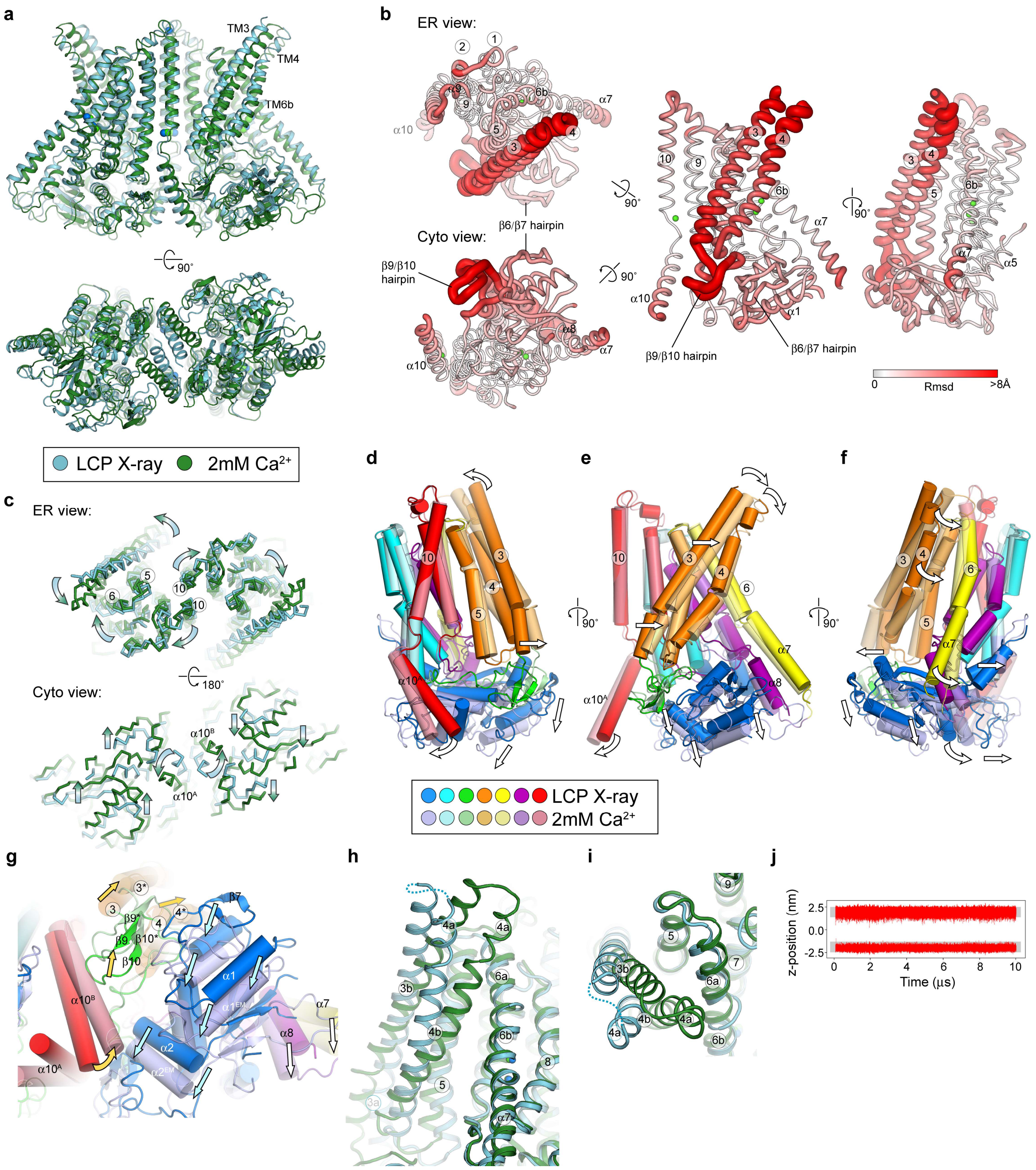

Supplementary Figure 12. Conformational changes between the crystal and cryo-EM structures. **a** Overall comparison of the LCP X-ray (blue) and 2 mM Ca^{2+} cryo-EM structure (green). **b** Global conformational differences between LCP X-ray and 2 mM Ca^{2+} cryo-EM monomer structures. Schematic representations of the rms deviation (rmsd) in mainchain atomic positions mapped onto the monomer structure. Monomers from each structure were superposed using all atoms with LSQKAB (CCP4). The monomer is viewed from either the luminal or cytoplasmic face (left), perpendicular to the membrane normal (middle) and onto scramblase groove face (right). The thickness and colour of the tube reflects the magnitude of the rmsd between the two structures. **c** relative movements of the TM and cytoplasmic domains between the X-ray and cryo-EM structures when the dimers are superimposed. **d-g** Conformational differences between LCP X-ray (5OC9) and 2 mM Ca^{2+} structures. Schematic representation looking onto (**d**) dimer face, (**e**) perpendicular to the membrane normal, (**f**) the scramblase groove and (**g**) cytoplasmic domain. The structures were superposed in the context

of the full dimer using the scaffold region alone (see Methods). The X-ray structure is coloured in darker shades and the 2 mM Ca^{2+} cryo-EM structure in lighter shades. **h-i** Conformational differences in the vicinity of the scramblase groove between TM3-TM7 viewed (**h**) perpendicular to the groove (**i**) looking down the groove from the luminal face. **j** CG MD simulations, showing lack of movement of lipids across the membrane with the closed groove structure.

Supplementary Tables

Supplementary Table 1. TMEM16K and ER marker co-localisation. Parameters (PPC, M1, M2) obtained for various cell lines.

Cell type	Targets of primary antibodies	PPC	M1	M2
COS-7	TMEM16K-TEV-His_{10}-Flag/HRD1	0.83±0.01 (48)	0.90±0.02 (48)	0.85±0.02 (48)
COS-7	TMEM16K-TEV-His_{10}-Flag/CNX	0.69±0.02 (23)	0.75±0.04 (23)	0.73±0.05 (23)
U2OS	TMEM16K/KDEL	0.70±0.01 (42)	0.79±0.01 (42)	0.81±0.01 (42)

PPC, Pearson's coefficient; M1, Manders' M1 coefficient; M2, Manders' M2 coefficient. Data are presented as mean ± s.e.m. alongside the number of cells (n)

Supplementary Table 2. X-ray crystallography data, refinement and model statistics.

	TMEM16K LCP	TMEM16K VD	TMEM16K VD (STARANISO)
**PDB Code**	5OC9		6R65
**Data Collection**			
Beamline	I24	I24	
Space group	$P2_12_12_1$	$P2_12_12_1$	
Crystallisation conditions	0.1M MES pH 6.0, 0.1M NaCl, 0.1M $CaCl_2$, 30% PEG300	0.1M HEPES pH 7.0, 0.1M Ca Acetate, 22% PEG 400, 0.05 mM C12E9	
Cell dimensions			
a, b, *c* (Å)	59.15, 153.59, 218.78	113.16, 152.27, 153.70	
α, β, γ (°)	90, 90, 90	90, 90, 90	
Resolution [Å][1]	77 - 3.2 (3.20-3.28)[1]	78 - 3.5 (3.59-3.50)[1]	78 - 3.5 (3.61-3.5)
Resolution limits [Å][2,3,4]	4.00, 3.43, 3.20	3.66, 5.86, 3.63	3.5, 5.2
	(3.68, 3.25, 3.20)[3]	(3.50, 5.16, 3.50)[3]	
Nominal Resolution [Å][5]	3.39	3.97	na
$CC_{1/2}$	0.990 (0.535)	1.00 (0.352)	1.00 (0.508)
R_{meas}	0.318 (1.398)[1]	0.201 (2.200)[1]	0.178 (0.96)
R_{pim}	0.139 (0.610)	0.103 (1.104)	0.12 (0.651)
$I / \sigma I$	5.9 (1.4)[1]	5.6 (0.8)[1]	7.1 (1.7)
Completeness [%]	99.9 (99.9)[1]	99.6 (99.8)[1]	76.4 (44.1)
Redundancy	5.1 (5.2)[1]	3.7 (3.8)[1]	3.6 (3.5)
**Refinement**			
Resolution (Å)	76.79 – 3.2		40 – 3.5
No. reflections (free)	33824 (1699)		23883 (1191)
R_{work} / R_{free}	22.9 / 24.5		23.7 / 24.9
No. atoms			
Protein	9806		9642
Other	332		6
B-factors (Å^2)			
Protein	57.76		92.19
Other	54.04		80,44
R.m.s. deviations			
Bond lengths (Å)	0.008		0.008
Bond angles (°)	0.87		0.90

[1] Values in parentheses are statistics for highest resolution shell
[2] Anisotropic resolution limits along each of the three principal directions as defined by AIMLESS based on Mn (I/sd(I)) > 2
[3] Values in parentheses are resolution limits in each direction based on half dataset correlation > 0.5 ($CC_{1/2}$).
[4] Values for STARANISO are best and worst diffraction limit after anisotropic truncation.
[5] Nominal resolution is defined based on overall Mn (I/sd(I)) > 2 as estimated by AIMLESS.

Supplementary Table 3. Cryo-EM data, refinement and model statistics.

**Cryo-EM data**	2 mM Ca^{2+}	430 nM Ca^{2+}	Ca^{2+}-free
**PDB code**	6R7X	6R7Y	6R7Z
**EMDB code**	EMD-4746	EMD-4747	EM-4748
**Data Collection:**			
Voltage (kV)	300	300	300
Defocus range (µm)	-1.25 to -3.0	-1.0 to -3.0	-1.0 to -3.0
Pixel size (Å)	0.822	0.822	0.822
Electron dose ($e^-/Å^2$)	51	56.5	56.5
Dose rate ($e^-/Å^2/s$)	6.36	7.06	7.06
Number of micrographs (used)	4095 (3421)	1975 (1446)	2083 (2038)
Particles (initial)[1]	300992	102669	143308
Particles (final, %)	70940 (24)	53880 (52)	23607 (16)
**Reconstruction:**			
Number of particles	70940	53880	23607
Symmetry	C2	C2	C2
Box size (pixels)	228	228	200
Resolution (unmasked, Å)[2]	3.75	4.36	6.3
Resolution (masked, Å)[2]	**3.47**	**4.2**	**5.14**
Map sharpening *B*-factor ($Å^2$)	-112	-176	-371
**Model composition:**			
Non-hydrogen atoms	10630	10062	6762
Protein residues	1278	1260	1228
**Refinement:**			
Resolution (Å)	3.5	4.2	5.1
Map sharpening factor ($Å^2$)	-112	-176	-150
Fourier Shell Correlation (FSC)[3]	0.8305	0.84694	0.8876
d_FSC (Map vs. Model)[4]	3.33 (3.47)	3.97 (4.15)	4.91 (6.30)
**Rms deviations:**			
Bonds (Å)	0.004	0.01	0.004
Angles (°)	0.710	1.034	0.80
**Molprobity validation:**			
Clashscore, all atoms	3.15	3.88	0.77
Molprobity score	1.22	1.49	0.84
Ramachandran Plot (% favoured)	97.45	97.28	97.53
Ramachandran Plot (% allowed)	2.39	2.56	2.47
Ramachandran Plot (% outliers)	0.16	0.16	0.0

[1] Particles after one cycle of 2D classification to remove non-particles
[2] Based on FSC 0.143 threshold in RELION
[3] CC_mask from phenix_real_space_refine
[4] d_fsc resolution estimate FSC=0.143 (FSC=0.5) from phenix.mtriage

Supplementary Table 4. List of primers used in this study.

Primer purpose		Primer sequence (5’ to 3’)
WT TMEM16K	Forward	TTAAGAAGGAGATATACTATGAAAGTGACCTTATCAGCTTTGGA
WT TMEM16K	Reverse	GATTGGAAGTAGAGGTTCTCTGCGGTTGCCTTCTCCTTCCCG
D615N	Forward	ATAAGCCACGGCATATCCAGATGAAACTAGCCAGAC
D615N	Reverse	TAGGTATGGCAAATGCAAGTATAAACTTTAAAGCCAG

Note: The same primers were used for cloning into vectors for expression in Sf9 and Expi239F cells.
