# Supplementary material for: The structural basis of lipid scrambling and inactivation in the endoplasmic reticulum scramblase TMEM16K
Source: Nat Commun. 2019 Sep 2;10:3956. doi: 10.1038/s41467-019-11753-1 (PMC6718402; doi:10.1038/s41467-019-11753-1)
Supplement: Supplementary file 3 — Description of Additional Supplementary Files [file 41467_2019_11753_MOESM3_ESM.pdf]

Description of Additional Supplementary Files

File name: Supplementary Movie 1

Description: Conformational morph between the LCP X-ray and the 2 mM Ca^{2+} cryo-EM structure. Structures are coloured based on the three main structural units identified by DYNDOM torsional analysis (static scaffold TM region (red), cytoplasmic domain (blue) and β9-β10-TM3-5-α10 unit (orange)). Coordinate morphing and the movie creation were carried out using UCSF Chimera.
