# Supplementary material for: The structural basis of lipid scrambling and inactivation in the endoplasmic reticulum scramblase TMEM16K
Source: Nat Commun. 2019 Sep 2;10:3956. doi: 10.1038/s41467-019-11753-1 (PMC6718402; doi:10.1038/s41467-019-11753-1)
Supplement: Supplementary file 5 — Reporting Summary [file 41467_2019_11753_MOESM5_ESM.pdf]

nature research

Corresponding author(s): Prof. Elisabeth Carpenter

Reporting Summary

Nature Research wishes to improve the reproducibility of the work that we publish. This form provides structure for consistency and transparency in reporting. For further information on Nature Research policies, see Authors & Referees and the Editorial Policy Checklist.

Statistical parameters

When statistical analyses are reported, confirm that the following items are present in the relevant location (e.g. figure legend, table legend, main text, or Methods section).

n/a	Confirmed	
☐	☒	The exact sample size (n) for each experimental group/condition, given as a discrete number and unit of measurement
☐	☒	An indication of whether measurements were taken from distinct samples or whether the same sample was measured repeatedly
☐	☒	The statistical test(s) used AND whether they are one- or two-sided<br>*Only common tests should be described solely by name; describe more complex techniques in the Methods section.*
☐	☒	A description of all covariates tested
☐	☒	A description of any assumptions or corrections, such as tests of normality and adjustment for multiple comparisons
☐	☒	A full description of the statistics including central tendency (e.g. means) or other basic estimates (e.g. regression coefficient) AND variation (e.g. standard deviation) or associated estimates of uncertainty (e.g. confidence intervals)
☐	☒	For null hypothesis testing, the test statistic (e.g. F, t, r) with confidence intervals, effect sizes, degrees of freedom and P value noted<br>*Give P values as exact values whenever suitable.*
☒	☐	For Bayesian analysis, information on the choice of priors and Markov chain Monte Carlo settings
☒	☐	For hierarchical and complex designs, identification of the appropriate level for tests and full reporting of outcomes
☐	☒	Estimates of effect sizes (e.g. Cohen's d, Pearson's r), indicating how they were calculated
☐	☒	Clearly defined error bars<br>*State explicitly what error bars represent (e.g. SD, SE, CI)*

Our web collection on statistics for biologists may be useful.

Software and code

Policy information about availability of computer code

Data collection	Confocal Microscopy Imagery was collected using Zen 2012 (Blue Edition) software. CryoEM data was collected using EPU software on a K2 Summit detector (Gatan). Crystallographic diffraction data was collected using standard synchrtron control software at Diamond Light Source I24.
Data analysis	Statistical data for functional assays were analysed using Graphpad Prism 7, SSPS Statistics or Microsoft Excel 2013. Crystallography data processing was performed using XDS with additional software from the Collaborative Computational Project No. 4 (CCP4) software suite or PHENIX (v1.13). Structural models were built using Coot (v0.8.9.1) with model refinement performed by BUSTER (v2.10.3). Initial CryoEM data was processed using the RELION software suite with further refinement using MotionCor2, CTFFind (v4.0) with automated particle picking using Gautomatch. Local resolution estimation was performed using blocres in BSOFT. Functional data was analysed used Graphpad Prism, SPSS and Microsoft Excel. Confocal microscopy image analysis was performed using the FIJI plugin package of ImageJ. Molecular Dynamic simulations were run using GROMACS. Structure figures were generated using Pymol or UCSF Chimera.

For manuscripts utilizing custom algorithms or software that are central to the research but not yet described in published literature, software must be made available to editors/reviewers upon request. We strongly encourage code deposition in a community repository (e.g. GitHub). See the Nature Research guidelines for submitting code & software for further information.

Data

Policy information about availability of data

All manuscripts must include a data availability statement. This statement should provide the following information, where applicable:

- Accession codes, unique identifiers, or web links for publicly available datasets
- A list of figures that have associated raw data
- A description of any restrictions on data availability

X-ray crystallography data is deposited in the Protein Data Bank (PDB) with the following accession codes 5OC9 and 6R65 for the LCP and vapour diffusion structures respectively. The cryo-EM density maps and the atomic coordinates have been deposited in the Electron Microscopy Data Bank (EMDB) and in the PDB under the following accession codes: EMD-4746 (6R7X), EMD-4747 (6R7Y) and EMD-4748 (6R7Z), for 2 mM Ca2+, 430 nM Ca2+ and Ca2+-free forms respectively. Source data is available in a supplementary file for Fig. 1b, 1d, 1e and 1f, Fig 2b-f, Fig. 2h, Supplementary Figure 1b and Supplementary Figure 3a-f.

Field-specific reporting

Please select the best fit for your research. If you are not sure, read the appropriate sections before making your selection.

☒ Life sciences ☐ Behavioural & social sciences ☐ Ecological, evolutionary & environmental sciences

For a reference copy of the document with all sections, see nature.com/authors/policies/ReportingSummary-flat.pdf

Life sciences study design

All studies must disclose on these points even when the disclosure is negative.

Sample size	This work does not involve the use of animals or human data, so we did not use any statistical methods to determine suitable sample sizes.
Data exclusions	No data was excluded from the electrophysiology or functional assays.
Replication	The electrophysiology and scramblase assays were performed on at least two separate purifications, which represent two biological repeats. The numbers of replicates are given for each experiments and they were always at least six repeats per sample.
Randomization	This is not relevant to our study because we were not comparing two sets of individuals who had been selected as control or sample groups.
Blinding	As we were not comparing animal or human populations with individuals assigned to groups, it was not relevant to do double blind tests.

Reporting for specific materials, systems and methods

Materials & experimental systems

n/a	Involved in the study	
☐	☒	Unique biological materials
☐	☒	Antibodies
☐	☒	Eukaryotic cell lines
☒	☐	Palaeontology
☒	☐	Animals and other organisms
☒	☐	Human research participants

Methods

n/a	Involved in the study	
☒	☐	ChIP-seq
☒	☐	Flow cytometry
☒	☐	MRI-based neuroimaging

Unique biological materials

Policy information about availability of materials

Obtaining unique materials	All the biological samples including cell lines and antibodies used in this work are commercially available, apart from the U2OS/Flip-in system, which is described in the manuscript as "U2OS FlpIn/T-Rex cells (kind gift of M. Gyrd-Hansen, Ludwig Institute, validated and described in PMID:23806334 and 30578317", with the relevant references. The mammalian expression vectors for BacMam expression are available upon request from the Frederick Boyce's lab, where the system was created and it is available, as stated in the manuscript "The BacMam vector backbone (pHTBV1.1) was kindly provided by Professor Frederick Boyce, Massachusetts General Hospital, Cambridge, MA". The SGC has a policy of making all its expression systems available for any published structures, so the vectors and expression plasmids are available in Addgene. So all materials are either available from commercial sources, from the SGC upon request or from the originators of the systems.

Antibodies

Antibodies used	Primary antibodies:<br>Anti-FLAG (Sigma, clone M2, F1804, mouse monoclonal)<br>anti-calnexin (CNX, Enzo Life Sciences, Adi_Spa-860, rabbit polyclonal)<br>anti-Hrd1 (Abcam, EP7459, rabbit polyclonal)<br>anti-TMEM16K (Sigma, HPA051569, rabbit polyclonal)<br>anti-KDEL (Enzo Life Sciences, 10C3, ADI-SPA-827, mouse monoclonal).<br><br>Also secondary antibodies:<br>secondary antibodies: anti-mouse IgG-Alexa 546 (Thermo Fisher)<br>anti-rabbit IgG-Alexa 488 (Thermo Fisher)
Validation	• Anti-FLAG: Validated for IF of human cells by the manufacturer, Sigma.<br>• anti-calnexin: Validated for IF of human and monkey cells by the manufacturer, Enzo Life Sciences.<br>• anti-Hrd1: Validated for IF of mammalian cells by the manufacturer, Abcam.<br>• anti-TMEM16K: Validated for IF in human and monkey cell lines in Supplementary Fig. 1. Also tested in a Western Blot with purified TMEM16K and cell lysates from HEK293F cells with and without overexpression of TMEM16K. (Supplementary Fig. 1d, this work).<br>• anti-KDEL: Validated for IF of mammalian cells by the manufacturer, Enzo Life Sciences.<br><br>Secondary Antibodies:<br>• anti-mouse IgG-Alexa 546 (Thermo Fisher): Validated for IF of human cells by the manufacturer<br>• anti-rabbit IgG-Alexa 488 (Thermo Fisher): Validated for IF of human cells by the manufacturer

Eukaryotic cell lines

Policy information about cell lines

Cell line source(s)	Spodoptera frugiperda (Sf9) insect cells and Expi239F from Thermo Fisher catalogue number 11496015 and Expi293F cells (Thermo-Fisher Scientific, Cat. No. A14527) were used for TMEM16K expression.<br><br>HEK-293T were obtained from the ATCC collection, reference ATCC CRL-1573<br>COS-7 cells were obtained from the ATCC collection, reference ATCC CRL-1651<br>U2OS FlpIn/T-Rex cells (kind gift of M. Gyrd-Hansen, Ludwig Institute, validated and described in PMID:23806334 and 30578317
Authentication	Standard laboratory model overexpression strains purchased from Thermo Fisher and the ATCC collection were used. These cell lines undergo quality control before dispatch. Cells were passaged a limited number of times before a new batch from the manufacturer was employed. Cells were monitored by regular visual inspection.
Mycoplasma contamination	our U2OS and COS-7 cells were routinely tested for mycoplasma contamination
Commonly misidentified lines<br>(See ICLAC register)	n/a
